# Are Immune Modulating Single Nucleotide Polymorphisms Associated with Necrotizing Enterocolitis?

**DOI:** 10.1038/srep18369

**Published:** 2015-12-16

**Authors:** Ashanti L. Franklin, Mariam Said, Clint D. Cappiello, Heather Gordish-Dressman, Zohreh Tatari-Calderone, Stanislav Vukmanovic, Khodayar Rais-Bahrami, Naomi L. C. Luban, Joseph M. Devaney, Anthony D. Sandler

**Affiliations:** 1 Division of General and Thoracic Surgery, Children’s National Health System, 111 Michigan Ave NW, Washington, DC 20010; 2 Division of Neonatology, Children’s National Health System, Washington, DC, Department of Pediatrics, The George Washington School of Medicine and Health Sciences, 111 Michigan Ave NW, Washington, DC 20010; 3 Children’s Research Institute, Children’s National Health System, Washington, DC, Department of Pediatrics, The George Washington School of Medicine and Health Sciences, 111 Michigan Ave NW, Washington, DC 20010; 4 Sheikh Zayed Institute, Children’s National Health System, Washington, DC, Department of Pediatrics, The George Washington School of Medicine and Health Sciences, 111 Michigan Ave NW, Washington, DC 20010; 5 Department Laboratory Medicine, Children’s National Health System Washington, DC, Department of Pediatrics, The George Washington School of Medicine and Health Sciences, 111 Michigan Ave NW, Washington, DC 20010; 6 Department of Genetic Medicine, Children’s National Health System, 111 Michigan Ave NW, Washington, DC 20010; 7 Division of General and Thoracic Surgery, Children’s National Health System, Washington, DC, Department of Pediatrics, The George Washington School of Medicine and Health Sciences, 111 Michigan Ave NW, Washington, DC 20010

## Abstract

Necrotizing enterocolitis (NEC) is a devastating gastrointestinal emergency. The purpose of this study is to determine if functional single nucleotide polymorphisms (SNPs) in immune-modulating genes pre-dispose infants to NEC. After Institutional Review Board approval and parental consent, buccal swabs were collected for DNA extraction. TaqMan allelic discrimination assays and BglII endonuclease digestion were used to genotype specific inflammatory cytokines and TRIM21. Statistical analysis was completed using logistic regression. 184 neonates were analyzed in the study. Caucasian neonates with IL-6 (rs1800795) were over 6 times more likely to have NEC (p = 0.013; OR = 6.61, 95% CI 1.48–29.39), and over 7 times more likely to have Stage III disease (p = 0.011; OR = 7.13, (95% CI 1.56–32.52). Neonates with TGFβ-1 (rs2241712) had a decreased incidence of NEC-related perforation (p = 0.044; OR = 0.28, 95% CI: 0.08–0.97) and an increased incidence of mortality (p = 0.049; OR = 2.99, 95% CI: 1.01 – 8.86). TRIM21 (rs660) was associated with NEC-related intestinal perforation (p = 0.038; OR = 4.65, 95% CI 1.09–19.78). In premature Caucasian neonates, the functional SNP IL-6 (rs1800795) is associated with both the development and increased severity of NEC. TRIM21 (rs660) and TGFβ-1 (rs2241712) were associated with NEC- related perforation in all neonates in the cohort. These findings suggest a possible genetic role in the development of NEC.

Necrotizing enterocolitis (NEC) is a leading cause of morbidity and mortality among preterm neonates^1^,^2^. Its prevalence has increased over the last 30 years as advances in neonatal critical care have led to improved survival of more preterm neonates. In fact, a recent study looking at mortality among extremely premature neonates between 2000–2011, noted that although overall mortality among this population has declined, deaths related to NEC have increased^3^. There is an overall mortality rate of 20–30% in infants with NEC and approaches 100% in neonates with pan-intestinal disease (NEC totalis)^4^. The onset of NEC is variable, and is inversely proportional to gestational age^5^. Signs and symptoms of the disease are often non-specific and require a high index of suspicion. The diagnosis of NEC is made using specific criteria as classified by the Modified Bell Staging (Table 1)^6^,^7^.

In the preterm infant, the gastrointestinal host defense is impaired, thereby increasing the risk of intestinal injury^8^. This inadequate host response allows for bacterial translocation and may lead to activation of an inflammatory cascade, resulting in global inflammatory effects and localized gastrointestinal inflammation^9^. Subsequently, several inflammatory mediators are released through activation of human toll like receptors (TLRs)^2^,^4^,^10^. Multiple inflammatory mediators are implicated in the pathogenesis of NEC^11^,^12^,^13^,^14^. Additionally, several studies have demonstrated that infants with NEC have elevated serum levels of inflammatory cytokines^15^,^16^,^17^. This finding however has no bearing on etiology, as it may simply be a pathologic response to the inflammatory process already underway.

Few studies have interrogated the role of genetics in the pathogenesis of NEC^18^,^19^,^20^. In an attempt to study whether an exaggerated inflammatory response has a causal relationship to developing NEC, we sought to identify genetic markers of the disease. In this study, we examined select single nucleotide polymorphisms (SNPs) among various inflammatory genes. Genes of interest were selected based on known associations between inflammatory mediators and NEC^12^,^13^,^14^,^21^. SNPs are naturally occurring single base pair changes in the genome. SNPs can alter the translation of their related proteins, altering function and disease processes. SNPs in TNFα and IL-6 are associated with altered levels of cytokines in serum, worsened outcomes in trauma patients, and various other pathologies^22^,^23^,^24^,^25^. We hypothesize that SNPs in genes encoding various inflammatory modulators may be associated with the exuberant gastrointestinal inflammation observed in NEC.

## Methods

### Patient Selection

This study was approved by the Institutional Review Board (IRB) at Children’s National Health System (Washington, DC, USA). All experiments were conducted according to institutional and IRB guidelines and regulations. Parental and/or guardian consent was obtained in accordance with our institutional policies. This was a prospective cohort study, in which premature neonates were recruited from the Neonatal Intensive Care Unit (NICU) at Children’s National Health System. Neonates <32 weeks gestation and any infant with the diagnosis of NEC (>Bell stage II) regardless of gestational age were included in the study. Controls were infants <32 weeks without the diagnosis of NEC (>Bell stage II). Neonates with congenital heart disease (except patent ductus arteriosus), major congenital or chromosomal disorders, and/or inborn errors of metabolism were excluded. Infants with complex congenital heart disease (CCHD) were excluded from the study to allow us to more clearly interrogate the role of genetics in NEC. Infants with CCHD, particularly those with left-sided obstructive cardiac lesions, have an increased risk of developing NEC due to potential intestinal hypoperfusion, thought to result from episodes of inadequate cardiac output, shock and diastolic flow reversal in the abdominal aorta^26^.

### Sample Collection and Preparation

Cytological brushes were used to swab the buccal mucosa and obtain neonatal DNA using methodology previously validated by our group^27^. Enrolled subjects were nil per os (NPO) at the time of buccal swab collection. DNA was extracted from buccal swab specimens using Qiagen Buccal Cell Kit (Qiagen Sciences, Germantown, MD). The buccal cell extraction kit was modified for a cell lysate volume of 900μl^27^. After cell lysis, genomic DNA was extracted with 5 μl proteinase k and isopropanol/ethanol precipitation.

### Genotyping of TRIM-21

#### PCR amplification–AccuPrime Taq DNA polymerase system (Invitrogen – Grand Island, NY)

100–200 ng of genomic DNA was amplified using the provided protocol for a 50 uL reaction. The PCR cycle was optimized for our primers−94 °C × 2 min, 94 °C × 30 sec, 58 °C × 30 sec, 68 °C × 30 sec for 35 cycles, 68 °C × 6 min, 10 °C∞. Forward primer sequence: 5′–CTG TAC ATC CAC AGT GAG C–3′. Reverse primer sequence: 5′–CAT CCC TTG TCA GAT GGA TAG–3′. A 3% agarose gel with NuSieve 3:1 was used to assess the presence of PCR products.

#### Allelic discrimination of TRIM21 with BglII (New England BioLabs – Ipswich, MA) digestion

**Figure 1:**
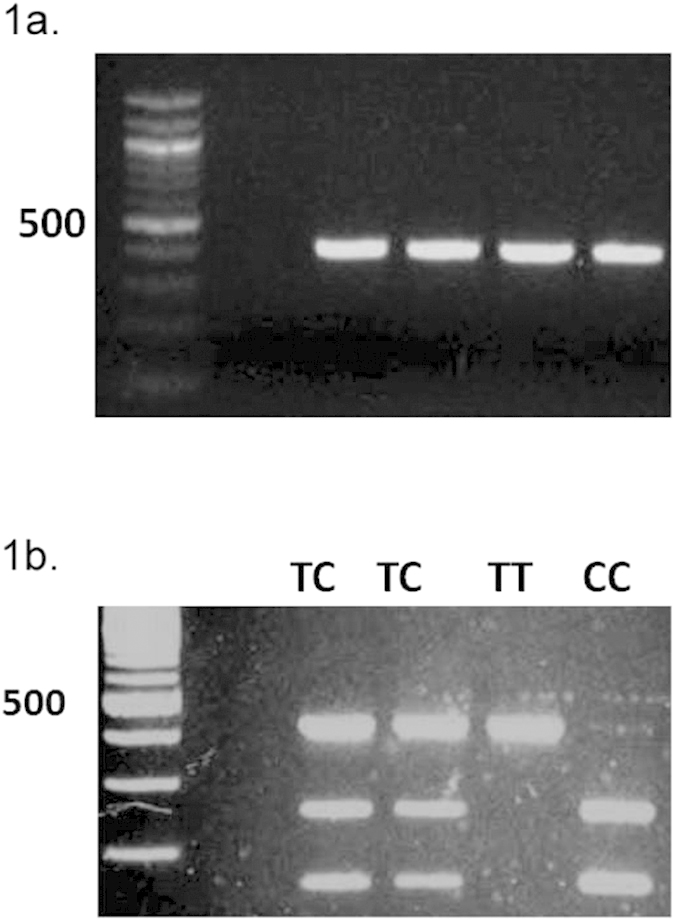
PCR Amplification and BglII endonuclease digestion (**a**) PCR amplification of TRIM21 rs660 with a product at 420 bp. (**b**) Rs660 there is a nucleotide alteration from T to C that will alter the site of BglII endonuclease digestion with resultant products at 255 and 165 bp.

5 uL of PCR product was used in a 50 uL reaction for restriction enzyme digestion with BglII^28^,^29^. Allelic discrimination was based on resultant banding patterns of digested fragments using gel electrophoresis as previously described^28^,^29^. Undigested PCR products produce 420 bp band (Fig. 1a). The rs660 SNP is a T → C polymorphism that alters the binding site of BglII. The resulting banding patterns correspond to the following genotypes: TT (420 bp band), TC (420 bp, 255 bp, 165 bp) and CC (255 bp, 165 bp) (Fig. 1b).

### Genotyping of inflammatory cytokine genes

Genotyping of SNPs from the following inflammatory cytokines IL-1B (rs16944), IL-6 (rs1800795), IL-12 (rs3212227), NOS3 (rs1800779), PXR (rs6785049), TGFβ-1 (rs2241712), TLR4 (rs4986790), and TNFα (rs1800629) were completed utilizing TaqMan^®^ Gene Expression system (Life Technologies, Grand Island, NY). Assays for IL-1B (C___1839943_10), IL-6 (C___1839697_20), IL-12 (C___2084293_10), NOS3 (C___7599687_1_), PXR (C__29280426_10), TGFβ-1 (C__15873873_10), TLR4 (C__11722238_20), and TNFα (C___7514879_10) were purchased from Life Technologies. Genotypes were obtained using a TaqMan^®^ allelic discrimination assay that employs the 5′ nuclease activity of Taq polymerase to detect a fluorescent reporter signal generated during PCR reactions. The PCR reactions contained 20 ng/μl of DNA, 900 nM primers, 200 nM probes, and TaqMan^®^ Universal PCR Master Mix, No AmpErase^®^ UNG (Applied Biosystems, Foster City, CA) in a final volume of 8 μl.

### Statistics

Statistical analysis was completed using a dominant model, comparing wild-type homozygous to both heterozygous and homozygous rare allele groups combined. This assumes that carrying at least one copy of the variant allele confers increased or decreased risk of disease^30^,^31^. Primary outcome measures included the presence of NEC, severity of disease (Bell’s stage III, NEC totalis), NEC-related bowel perforation, and mortality. Hardy-Weinberg equilibrium (HWE) was determined using a χ^2^ test to compare the observed genotype frequencies to those expected under Hardy-Weinberg equilibrium (HWE). Comparisons of genotype frequencies and outcomes between Blacks and Caucasians were performed using χ^2^ tests. Fisher’s exact test was used to perform crude comparisons between outcomes in Blacks versus Caucasians. Logistic regression or exact logistic regression was used depending on the number of subjects available for analysis. Ordinal logistic regression was utilized to compare severity of disease. Significance level was set at p < 0.05 and Stata V13 was (College Station, TX) used for all statistical analysis.

The analysis was performed using a two-stage analysis. Initially, associations between each outcome and each SNP were evaluated in the entire patient cohort with adjustment for race, ethnicity, gestational age, and gender. Only those outcomes/SNP pairs that showed either a statistically significant association (p < 0.05) or some evidence of a relationship (p < 0.15) were further investigated in racial/ethnic specific cohorts. All additional analyses included covariates for gestational age and gender.

## Results

**Figure 2:**
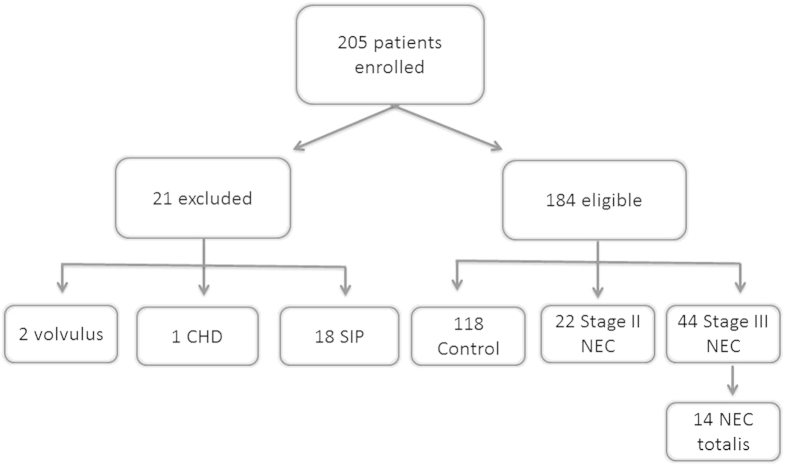
Study Cohort.

Two-hundred and five patients were enrolled in the study (Fig. 2). Twenty-one infants were excluded from analysis. One infant was found to have congenital heart disease (Tetralogy of Fallot). Two patients, initially diagnosed with NEC according to clinical presentation and radiographic evidence of pneumatosis, were found to have intestinal volvulus intraoperatively. Eighteen infants had a diagnosis of spontaneous intestinal perforation (SIP). SIP was distinguished from NEC based on radiographic imaging, clinical presentation, surgical findings, and medication history. Despite distinct differences between the two diseases, infants with SIP were excluded to not confound the data analysis.

The remaining 184 infants had a mean gestational age of 27.3 ± 3.3 weeks (22–40), and a mean birth weight of 1.02 ± 0.52. 101 (54.9%) enrolled subjects were male and 83 (45.1) female. There were 121 (65.8%) Black and 59 (32.1%) Caucasian subjects in this study (Table 2). Most patients were Non-Hispanic (81.4%). Assignation of race and ethnicity were determined by parental self-reporting. There were 118 controls and 66 patients with NEC (>Bell stage II). Of those neonates with NEC, 42 had surgical NEC, including bedside peritoneal drain placement and/or exploratory laparotomy (Table 3). 14 infants with Stage III disease were diagnosed with NEC totalis. The overall mortality rate was approximately 12% (n = 23), with over 70% of deaths in this cohort attributed to NEC (n = 17). In our study, Blacks and Caucasians of both Hispanic and Non-Hispanic ethnicity showed no significant differences in any of the measured outcomes (Table 4). There were substantial differences in allele frequencies between Caucasians and Blacks (supplemental Table 1), therefore, subsequent analyses were completed in Black and Caucasian subjects as individual cohorts. Individual analyses in the remaining racial categories (Asian or Other) were unable to be completed due to inadequate sample size. In the Black population, IL-12 (rs3212227) and PXR (rs6785049) were not in HWE. In Caucasians, IL1B (rs16944) and TLR4 (rs4986790) were not in HWE.

Initial analyses in the total cohort of patients showed some evidence of a relationship between the following outcomes and SNPs: IL-6 (rs1800795) with NEC and severity of disease, TGFβ-1 (rs2241712) with overall mortality and NEC perforation, and TRIM21 (rs660) with NEC perforation (Table 5). These associations were further analyzed in Blacks and Caucasians as separate cohorts.

In the total cohort, the G allele of TGFβ-1 (rs2241712) was associated with a significantly decreased incidence of NEC-related perforation (p = 0.044; OR = 0.28, 95% CI: 0.08–0.97) and a significantly increased incidence of overall mortality (p = 0.049; OR = 2.99, 95% CI: 1.01 – 8.86). These associations were not statistically significant in either of the race specific cohorts. In all subjects with NEC, TRIM21 (rs660) was associated with NEC-related intestinal perforation (p = 0.038; OR = 4.65, 95% CI 1.09–19.78). When evaluating TRIM21 in race specific cohorts, no association between NEC-related intestinal perforation and TRIM21 was found. In the total cohort, the C allele of IL-6 (rs1800795) trended toward an increased prevalence of NEC (p = 0.11; OR = 1.74, 95% CI: 0.88–3.42), as well as an increased severity of NEC (p = 0.054; OR = 2.06, 95% CI: 0.98–4.13). Analysis of Black neonates with IL-6 (rs1800795) showed no association between the incidence of NEC or severity of disease. Caucasian neonates with the C allele of IL-6 (rs1800795) were over 6 times more likely to have NEC than those with no C allele (p = 0.013; OR = 6.61, 95% CI 1.48–29.39) (Table 6). Caucasian neonates with the C allele of IL-6 (rs1800795) were also significantly more likely to have stage III than those without the C allele (p = 0.011; OR = 7.13, 95% CI 1.56–32.52) (Table 6).

In order to determine whether having multiple SNPs increased the risk of developing disease, a multi-SNP analysis was performed, with no significant results. A genetic risk score model to include IL-6, TRIM21 and TGFB1 was used, and did not find any evidence that the combined SNPs were better predictors of NEC (Table 7). This lack of a clear association may be in part due to the low numbers of individuals having none of the “risk” alleles for the SNPs, a limiting factor in this study.

## Discussion

This is one of the largest studies from the United States evaluating functional SNPs in immune modulating genes among neonates. While the exact pathogenesis of NEC remains unclear, it is widely accepted that an exuberant gastrointestinal inflammatory state accompanies this disease. In our study, over 30% of enrolled subjects had NEC. Of those with NEC, nearly 70% had surgical NEC and 21% had NEC totalis. The incidence of NEC in our study population is much greater than the national incidence, but similar to that reported from other freestanding children’s hospitals^32^. The increased prevalence and severity of disease in our study population is likely attributed to the fact that our NICU serves solely as a referral site for critically ill neonates in the region. In addition, our cohort is comprised largely of Blacks, (reflective of our overall NICU racial demographics), and may be related to the higher prevalence of NEC^33^. Contrary to the literature^33^, there was no significant difference in the incidence of NEC and severity of disease between race in our study population.

Multiple studies have shown that pro-inflammatory cytokines are increased in the serum and tissue of patients with NEC^15^,^16^,^17^,^34^. Additionally, several studies have demonstrated that neonates with NEC had a significantly higher elevation of serum inflammatory cytokines than infants with other gastrointestinal diseases^33^,^35^,^36^. Functional SNPs in inflammatory cytokines may be associated with an altered basal level expression of the cytokines in serum and disease^37^,^38^,^39^. This observation supports our hypothesis that functional SNPs that alter cytokine serum levels may predispose neonates to the excessive inflammation associated with NEC.

IL-6 is a proinflammatory cytokine that functions as a mediator of the acute phase response and fever. IL-6 (rs1800795) is located on chromosome 7 in the 5′ flanking promoter region and has a single nucleotide base substitution from a G to C allele at position 174^25^,^40^. The C allele is more common in Caucasians than in Blacks^41^ and is associated with an increased odds of developing NEC and having more severe disease. IL-6-174C is associated with an alteration in the promoter region that increases transcription of IL-6, which may predispose premature Caucasian infants to NEC. In the literature, there is conflicting evidence on the role of IL-6 expression in IL-6-174 genotypes. Kilpinen *et al.*^40^, found that neonatal monocytes stimulated with lipopolysaccharide (LPS) had higher IL-6 expression in neonates with IL-6-174C when compared to those with the G allele. Similarly, plasma expression of IL-6 was higher in neonates with IL-6-174C. Reiman *et al.*^24^, found that IL-6-174GG was associated with chorioamnionitis and IL-6-174CC was associated with neonatal sepsis. Reiman *et al* 24 did not find an association between IL-6- 174 and NEC, however this was likely due to very small sample size. Conversely, Fishman *et al.*^25^ found that the IL-6-174CC was associated with lower levels of IL-6 in the plasma of adults and may confer a protective benefit against juvenile chronic arthritis. Although we do not know the exact mechanism of IL-6-174C association with NEC in Caucasian neonates it is plausible that this SNP regulates IL-6 response to antigens in naïve neonatal cells leading to exuberant inflammatory response seen in infants with NEC^40^. Caucasian neonates with the C allele of IL-6 (rs1800795) were over 6 times more likely to have NEC. In addition, Caucasian infants with IL-6-174C were over 7 times more likely to have Stage III disease. This supports a hypothesis that an altered inflammatory state may be involved in the pathogenesis of NEC. Furthermore, our findings elucidate a possible genetic basis for the predisposition to NEC.

Our study revealed that infants with TRIM21 (rs660) were over 4 times more likely to have a NEC-related perforation. TRIM21 is a tripartite motif protein that is located on chromosome 11p15.5 in the non-coding region of the gene and leads to a single base alteration from a C to T allele^28^,^29^,^42^. Depending on the context, TRIM21 can have proinflammatory or anti-inflammatory actions^43^. TRIM21 can also serve as an intracellular receptor for antigen-antibody complexes internalized via cell surface receptors that infectious agents use to enter the cells. This intracellular interaction results in activation of intracellular immune pathways^44^. This activates the production of pro-inflammatory cytokines, which promote resistance to viruses and intracellular bacteria^45^. Conversely, in TRIM21 knock-out mice, TRIM21 functioned as a downregulator of NFκB and interferon signaling, highlighting its function as an anti-inflammatory protein^46^,^47^. A key question for understanding the potential role of TRIM21 in NEC is whether immune complexes formed by unknown antigens are involved in the pathogenesis of NEC. In either case, based on earlier studies by Tatari-Calderone *et al.*^28^ and Yoshimi *et al.*^42^ we predict that the rs660C allele is associated with lower levels of TRIM21. Therefore based on these and other studies^28^,^42^,^46^,^47^ it is possible that a decrease in TRIM21 as a result of the rs660C allele leads to an unregulated gastrointestinal pro-inflammatory response in neonates with NEC leading to the morbidity of NEC-related perforation.

TGFβ-1 (rs2241712) had a protective effect against NEC-related perforation in neonates with NEC, yet showed an increased incidence of overall mortality among the entire cohort. TGFβ*-*1 plays a role in immune modulation, anti-inflammation, and cell growth and differentiation. TGFβ-1 (rs2241712) is located on chromosome 19q in the 3′ UTR promoter region and leads to a single base change of G to A^48^. There is no data in the literature regarding the function of TGFβ-1 (rs2241712) or its effect on altering basal levels of TGFβ-1 in serum. It is thus not possible to explain the paradoxical effect of protection against intestinal perforation, yet increased overall mortality. More studies are needed to evaluate these seemingly paradoxical effects in NEC.

There are several limitations in this study. Although this study is one of the largest prospective cohort studies in the United States evaluating the genetic basis for NEC, our sample size is relatively small. Several outcome measures could not be adequately tested in the race specific cohorts due to an inadequate sample size, therefore the study may not be powered to detect a definitive association between SNPs in inflammatory cytokines and TRIM 21 and the diagnosis of NEC. The confidence intervals in our study were often wide in part due to the relatively small sample size. TGFβ-1 (rs2241712) and TRIM21 (rs660) were associated with NEC-related perforation in all subjects after adjusting for race and ethnicity, however this association was either not present or did not have an adequate sample size to test within individual racial groups making conclusions difficult. The large number of patients transferred for surgical management of NEC may have led to a selection bias. Additionally, to determine if there is an actual “cause and effect” relationship, inflammatory cytokines should be measured in plasma before and after the clinical presentation of NEC.

## Conclusion

Caucasian neonates with IL-6-174-C were over 6 times more likely to have NEC, and over 7 times more likely develop more severe disease. This study is a novel, large, and ongoing prospective cohort study evaluating the genetic predisposition to NEC. In addition, this study generates hypotheses for further genetic testing in neonates to determine predisposition to various neonatal diseases. Furthermore, association of multiple SNPs and NEC may allow for the development of a laboratory genetic test that could predict the risk/probability of premature neonates developing NEC. Identifying such an association would allow for preventative medical measures in these infants and provide insights into the pathogenesis of this disease, establishing the foundation for future therapies.

## Additional Information

**How to cite this article**: Franklin, A. L. *et al.* Are Immune Modulating Single Nucleotide Polymorphisms Associated with Necrotizing Enterocolitis? *Sci. Rep.* **5**, 18369; doi: 10.1038/srep18369 (2015).

## Supplementary Material

Supplementary Table 1

## Figures and Tables

**Table 1:** Modified Bell’s Staging Criteria (Kliegman *et al.*^12^).

Modified Bell’s Staging	Clinical Findings	Radiographical Findings	Gastrointestinal Findings
Stage I	Apnea, bradycardia, and temperature instability	Normal gas pattern or mild ileus	Mild abdominal distention, stool occult blood, gastric residuals
Stage IIA	Apnea, bradycardia, and temperature instability	Ileus with dilated bowel loops and focal pneumatosis	Moderate abdominal distention, hematochezia, absent bowel sounds
Stage IIB	Metabolic acidosis and thrombocytopenia	Widespread pneumatosis, portal venous gas, ascites	Abdominal tenderness and edema
Stage IIIA	Mixed acidosis, coagulopathy, hypotension, oliguria	Moderate to severely dilated bowel loops, ascites, no free air	Abdominal wall edema, erythema, and induration
Stage IIIB	Shock, worsening vital signs and laboratory values	Pneumoperitoneum	Bowel perforation

**Table 2:** Demographics.

Variable	Total *(n* = *184)*	Control *(n* = *118)*	NEC *(n* = *66)*	p-value
Male sex, *n (%)*	101 (54.9)	61 (51.7)	40 (60.6)	0.24
GA (weeks), mean ± SD	27.3 ± 3.3	27.1 ± 2.74	27.6 ± 4.14	0.33
Birth Weight (kg), mean ± SD	1.02 ± 0.52	0.99 ± 0.37	1.07 ± 0.51	0.22
*Race*				
Black, *n (%)*	121 (65.8)	75 (63.6)	46 (69.7)	0.42
Caucasian, *n* (*%*)	59 (32.1)	41 (34.8)	18 (27.3)	0.33
Asian, *n (%)*	2 (1.1)	0 (0.0)	2 (3.0)	0.12
Other, *n (%)*	2 (1.1)	2 (1.7)	0 (0.0)	0.53
*Ethnicity*				
Hispanic, *n (%)*	34 (18.5)	23 (12.5)	11 (6)	0.70
Mortality	23 (12.5)	6 (5.1)	17 (25.7)	<0.001

**Table 3:** NEC Demographics.

Variable	NEC cases (n = 66)
Surgical NEC	42 (63.6)
NEC Perforation	25 (37.9)
NEC-related Mortality	17 (25.8)
NEC *Totalis*	14 (21.2)
Black	46 (69.7)
Stage II	11 (23.9)
Stage III	35 (76)
*Totalis*	12 (34.3)*
Caucasian	18 (27.3)
Stage II	9 (50)
Stage III	9 (50)
*Totalis*	2 (22.2)*
Asian	2 (3)
Stage II	2 (100)

*indicates percentage of NEC stage III patients with given characteristic.

**Table 4:** Comparison of outcomes between Blacks and Caucasians.

Variable	Black (n = 121)	Caucasian (n = 59)	p-value
NEC, any stage, *n (%)*	46 (38)	18 (30.5)	0.41
NEC Stage III, *n (%)*	35 (28.9)	7 (11.9)	0.014
NEC *Totalis, n (%)*	12 (9.9)	2 (3.3)	0.15
NEC Perforation, *n (%)*	20 (16.5)	6 (10.2)	0.37
Overall Mortality, *n (%)*	18 (14.9)	4 (6.8)	0.15
NEC Mortality, *n (%)*	14 (11.6)	3 (5)	0.19

**Table 5:** Potential associations seen in the entire cohort.

SNP	Outcome	Genotype	Outcome			OR	p-value	95% CI
			No		Yes			
IL-6 (rs1800795)	NEC	GG	93		25	1.00		
		CG/CC	40		40	1.74	0.11	0.88– 3.42
IL-6 (rs1800795)	Overall mortality	GG	109		13	1.00		
		CG/CC	50		9	2.54	0.08	0.90–7.23
TGFβ-1 (rs2241712)	Overall mortality	AA	66		5	1.00		
		AG/GG	92		18	2.99	0.049	1.01–8.86
TGFβ-1 (rs2241712)	NEC perforation	AA	12		12	1.00		
		AG/GG	28		13	0.28	0.044	0.08–0.97
TRIM21 (rs660)	NEC perforation	TT	16		4	1.00		
		CT/CC	16		15	4.64	0.038	1.09–19.78
**SNP**	**Outcome**	**Genotype**	**Severity level**			**OR**	**p-value**	**95% CI**
			**0**	**2**	**3**			
IL-6 (rs1800795)	NEC severity	GG	83	14	26	1.00		
		CG/CC	34	8	17	2.06	0.054	0.98–4.13

**Table 6:** Association between IL6 and NEC in Hispanic and Non-Hispanic Caucasians.

SNP	Outcome	Genotype	Outcome			OR	p-value	95% CI
			No		Yes			
IL-6 (rs1800795)	NEC	GG	24		3	1.00		
		CG/CC	16		15	6.61	0.013	1.48–29.39
**SNP**	**Outcome**	**Genotype**	**Severity**			**OR**	**p-value**	**95% CI**
			**0**	**2**	**3**			
IL-6 (rs1800795)	NEC severity	GG	24	2	1	1.00		
		CG/CC	16	7	8	7.13	0.011	1.56–32.52

**Table 7:** Combined risk score with risk alleles for IL-6, TRIM21 and TGFβ-1.

Outcome	Race/ethnicity	Genotype risk score	Outcome		OR	p-vzalue	95% CI	P-value for trend over all risk scores
			No	Yes				
NEC	African-Americans	0	17	11	1.00			0.56
		1	37	22	0.94	0.90	0.37–2.41	
		2	16	10	0.98	0.97	0.32–2.96	
		3	5	1	0.33	0.34	0.03–3.28	
NEC	Caucasians	0	7	1	1.00			0.09
		1	21	7	1.77	0.63	0.17–18.62	
		2	11	7	2.72	0.42	0.23–31.38	
		3	2	3	9.97	0.12	0.53–185.69	
