# Supplementary material for: Are Immune Modulating Single Nucleotide Polymorphisms Associated with Necrotizing Enterocolitis?
Source: Sci Rep. 2015 Dec 16;5:18369. doi: 10.1038/srep18369 (PMC4680983; doi:10.1038/srep18369)
Supplement: Supplementary Table 1 [file srep18369-s1.doc]

Supplementary Information

*Allele Frequencies and Hardy-Weinberg Equilibrium (HWE)*

Are Immune Modulating Single Nucleotide Polymorphisms Associated with Necrotizing Enterocolitis?

Franklin, A.L., Cappiello, C.D., Gordish-Dressman, H., Tatari-Calderone, Z., Vukmanovic, S., Rais-Bahrami, K., Luban, N.L.C., Devaney, J.M., Sandler, A.D.

**Supplemental Table 1. Allele frequencies and HWE**

| **Race*** | **SNP** | **Observed frequencies** | | | **p(A)** | **p(B)** | **HWE p-value** |
| --- | --- | --- | --- | --- | --- | --- | --- |
| Black | TRIM21 (rs660)^ | 50 | 38 | 13 | 0.683 | 0.317 | 0.19 |
|  | TGFβ-1 (rs2241712)^ | 55 | 57 | 9 | 0.690 | 0.310 | 0.26 |
|  | TNFα (rs1800629)^ | 84 | 31 | 4 | 0.836 | 0.164 | 0.59 |
|  | NOS3 (rs1800779)^ | 83 | 30 | 6 | 0.824 | 0.176 | 0.15 |
|  | IL-6 (rs1800795)^ | 93 | 22 | 4 | 0.874 | 0.127 | 0.08 |
|  | IL-1B (rs16944)^ | 40 | 60 | 20 | 0.583 | 0.417 | 0.75 |
|  | IL-12 (rs3213337)^ | 55 | 58 | 4 | 0.718 | 0.282 | 0.015 |
|  | TLR4 (rs4986790) | 93 | 18 | 0 | 0.919 | 0.081 | 0.35 |
|  | PXR (rs6785049)^ | 56 | 37 | 20 | 0.659 | 0.341 | 0.004 |
| White | TRIM21 (rs660)^ | 11 | 25 | 8 | 0.534 | 0.466 | 0.35 |
|  | TGFβ-1 (rs2241712)^ | 15 | 29 | 12 | 0.527 | 0.473 | 0.77 |
|  | TNFα (rs1800629)^ | 44 | 12 | 2 | 0.862 | 0.138 | 0.32 |
|  | NOS3 (rs1800779)^ | 28 | 26 | 3 | 0.719 | 0.281 | 0.33 |
|  | IL-6 (rs1800795)^ | 27 | 24 | 7 | 0.672 | 0.328 | 0.64 |
|  | IL-1B (rs16944)^ | 16 | 20 | 21 | 0.456 | 0.544 | 0.027 |
|  | IL-12 (rs3213337)^ | 28 | 21 | 9 | 0.664 | 0.336 | 0.15 |
|  | TLR4 (rs4986790) | 50 | 3 | 1 | 0.954 | 0.046 | 0.006 |
|  | PXR (rs6785049)^ | 12 | 25 | 17 | 0.454 | 0.546 | 0.63 |

* Calculations include both Hispanic and Non-Hispanics

^ Significantly difference genotype distributions between Blacks and Whites
